# Budesonide/formoterol and formoterol provide similar rapid relief in patients with acute asthma showing refractoriness to salbutamol

**DOI:** 10.1186/1465-9921-7-13

**Published:** 2006-01-24

**Authors:** ED Bateman, L Fairall, DM Lombardi, R English

**Affiliations:** 1 University of Cape Town Lung Institute, Cape Town, South Africa; 2 Hospital Municipal de Rehabilitación Respiratoria 'María Ferrer', Buenos Aires, Argentina

## Abstract

**Background:**

To compare the efficacy and safety of budesonide/formoterol (Symbicort^®^) with formoterol (Oxis^®^) in the treatment of patients with acute asthma who showed evidence of refractoriness to short-acting β_2_-agonist therapy.

**Methods:**

In a 3 hour, randomized, double-blind study, a total of 115 patients with acute asthma (mean FEV_1 _40% of predicted normal) and a refractory response to salbutamol (mean reversibility 2% of predicted normal after inhalation of 400 μg), were randomized to receive either budesonide/formoterol (320/9 μg, 2 inhalations at t = -5 minutes and 2 inhalations at 0 minutes [total dose 1280/36 μg]) or formoterol (9 μg, 2 inhalations at t = -5 minutes and 2 inhalations at 0 minutes [total dose 36 μg]). The primary efficacy variable was the average FEV_1 _from the first intake of study medication to the measurement at 90 minutes. Secondary endpoints included changes in FEV_1 _at other timepoints and change in respiratory rate at 180 minutes. Treatment success, treatment failure and patient assessment of the effectiveness of the study medication were also measured.

**Results:**

FEV_1 _increased after administration of the study medication in both treatment groups. No statistically significant difference between the treatment groups was apparent for the primary outcome variable, or for any of the other efficacy endpoints. There were no statistically significant between-group differences for treatment success, treatment failure or patient assessment of medication effectiveness. Both treatments were well tolerated.

**Conclusion:**

Budesonide/formoterol and formoterol provided similarly rapid relief of acute bronchoconstriction in patients with asthma who showed evidence of refractoriness to a short-acting β_2_-agonist.

## Introduction

Patients presenting with symptoms of acute asthma are traditionally treated with short-acting β_2_-agonists [1,2]. Formoterol is a long-acting β_2_-agonist with a rapid onset of action, producing bronchodilation within 1–3 minutes of inhalation [3-5]. This effect is comparable with that of salbutamol [5], making formoterol suitable for the treatment of acute asthma. In this setting, formoterol has proved both safe and efficacious [6,7]. A large study performed in an emergency room setting by Boonsawat and colleagues [6] showed that high-dose formoterol was as rapid and effective as high-dose salbutamol in reversing bronchoconstriction in patients with severe asthma, but formoterol produced greater improvements than salbutamol in lung function over 4 hours. For this reason, formoterol has been licensed for use as both maintenance therapy and as an alternative to salbutamol and terbutaline for the relief of acute asthma symptoms.

Asthma patients experiencing acute symptoms may use their β_2_-agonist reliever medication repeatedly. This can result in downregulation of β_2_-receptors and consequent relative refractoriness to the bronchodilatory effects of this class of drug [8,9]. High doses of inhaled corticosteroid (ICS) have been reported to upregulate these receptors and restore β_2_-agonist responsiveness [10].

In order to study the potential of this favourable interaction, Pansegrouw [11] examined the use of combined ICS and short-acting β_2_-agonist treatment in patients with acute asthma who initially showed no response to β_2_-agonist therapy. It was reported that 'priming' patients with ICS before commencing nebulised β_2_-agonist treatment was more effective than therapy with the β_2_-agonist alone at improving features of the exacerbation, including lung function [11]. Although, to our knowledge, this study has not been repeated, it raises the prospect that budesonide and formoterol – which are now available together in a combination inhaler (Symbicort^®^; budesonide/formoterol) – may be more effective than formoterol given alone in treating patients with acute asthma who initially show no response to β_2_-agonist therapy. The present study was designed to compare the efficacy of budesonide/formoterol with that of formoterol for the treatment of patients with acute asthma who had evidence of relative refractoriness to the administration of a short-acting β_2_-agonist.

## Patients and methods

### Patients

Patients aged ≥12 years presenting with acute asthma were recruited from a total of 8 centres in Argentina, Mexico and South Africa. All patients were required to have asthma, as defined by the American Thoracic Society criteria (including symptoms of wheeze, episodic cough, and dyspnea) [12], with a pre-bronchodilator forced expiratory volume in 1 second (FEV_1_; measured on arrival in the acute setting) ≥30% and ≤55% of predicted normal. In addition, patients had to have a relative lack of reversibility, as demonstrated by their FEV_1 _improving by 8% or less of predicted normal, 10 minutes after receiving salbutamol 400 μg from a pressurised metered-dose inhaler.

Exclusion criteria included: acute severe asthma (defined as an inability to generate an FEV_1 _value, an FEV_1 _of less than 30% predicted, or asthma requiring transfer to an intensive care unit on initial assessment); use of ICS within the 8 hours preceding the baseline measurements; receipt of oral or other systemic steroids in the 48 hours before the baseline measurements; β-blocker therapy (including eye drops); any significant disease or concomitant disorder; and known sensitivity to the study medication or lactose. Patients ≥45 years of age with a history of ≥10 pack-years of smoking were also excluded from the study.

During the treatment period, patients were not permitted to receive any asthma medication other than the investigational product, although oxygen therapy was allowed. Other medication considered necessary for the patient's safety and well-being, and early withdrawal from the study, were permitted at the discretion of the investigator.

### Study design

This was a 180 minute, double-blind, double-dummy, randomized, parallel-group multicentre study (Study 0693). The study was performed in accordance with the ethical principles in the Declaration of Helsinki, Good Clinical Practice guidelines, in addition to applicable local regulatory requirements, and the protocol was approved by local ethics review boards. Before any procedure relating to the study was performed, written informed consent was obtained from the patients and, where applicable, from the patient's parent or legal guardian.

**Figure 1:**
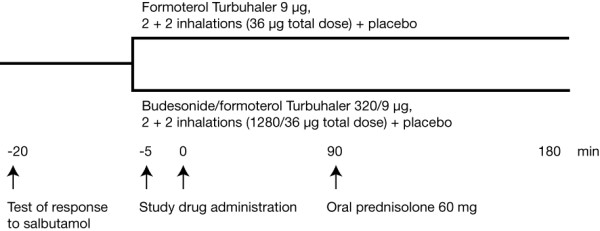
Study design.

The study design is shown in Figure 1. After the test of response to salbutamol (Ventolin^®^, Glaxo Wellcome, UK; 100 or 200 μg [total dose 400 μg], administered using a pressurised metered-dose inhaler with a Volumatic spacer [Allen & Hanburys, UK]), eligible patients received either budesonide/formoterol (Symbicort^® ^Turbuhaler^®^, AstraZeneca, Sweden; 320/9 μg, 2 inhalations at t = -5 minutes and a further 2 inhalations at t = 0 minutes [total dose 1280/36 μg], plus 2 inhalations of formoterol placebo containing lactose at t = -5 minutes and at t = 0 minutes) or formoterol (Oxis^® ^Turbuhaler^®^, AstraZeneca, Sweden; 9 ug, 2 inhalations at t = -5 minutes and a further 2 inhalations at t = 0 minutes [total dose 36 μg], plus 2 inhalations of budesonide/formoterol placebo containing lactose at t = -5 minutes and at t = 0 minutes). Oral prednisolone (Approved Prescription Services, UK; 5 mg per tablet, 12 tablets [total dose 60 mg]) was administered to all patients 90 minutes after they received the second dose of study medication.

### Efficacy assessments

The primary efficacy variable was the average FEV_1 _from the first intake of study drug to the measurement at 90 minutes. The following were assessed as secondary efficacy variables: the change in FEV_1 _at t = 3, 15, 60, 90 and 180 minutes after the last intake of study medication; the change in respiratory rate at 180 minutes; the proportion of patients with treatment success at 90 and 180 minutes; the proportion of patients with treatment failure at 180 minutes; and the effectiveness of the study medication at 3 and 15 minutes.

FEV_1 _was measured by spirometry according to European Respiratory Society (ERS) recommendations [13]. Respiratory rate was counted for 1 minute at baseline (t = -15 minutes) and at 15, 30, 60, 90, 120, and 180 minutes after the last intake of study medication. Treatment success was judged by the investigator and defined in terms of a patient being fit for immediate discharge at 90 and 180 minutes. If treatment was considered successful at 90 minutes, the patient still remained in the study for the 180-minute period. Treatment failure was defined as a requirement for additional asthma treatment and/or hospitalization because of asthma during the timeframe of the study. A subjective assessment of the effectiveness of the study medication was determined by asking the patients whether or not they considered the medication to be effective. An uncertain response was recorded as 'no'.

### Safety assessments

The incidence, severity and type of adverse events occurring during the study were recorded. Deterioration of asthma was not recorded as an adverse event unless it caused the patient to withdraw from the study or was classed as serious by the investigator. A serious adverse event was defined as an event that: caused death or was immediately life-threatening; required inpatient care or prolongation of hospitalization; resulted in persistent or significant disability or incapacity; or required medical intervention to prevent one of these outcomes.

Measurements of serum potassium, pulse rate, blood pressure, electrocardiogram (ECG) variables, and oxygen saturation were also used to assess safety. The outcome variables for serum potassium were the minimum and average values after administration. Blood samples for the determination of this variable were obtained at baseline (t = -15 minutes) and 30, 90 and 180 minutes after administration of the last dose of study medication.

### Statistical methods

A sample size of 50 patients per group was required to have an 80% chance of detecting a difference of 12% between treatments in average FEV_1 _from the first intake of study drug to the 90 minute measurement (5% significance level, *t*-test, two-sided alternative hypothesis).

Efficacy analyses were performed on the full analysis set; all patients who received at least one dose of study medication were included in the safety analysis. Changes in FEV_1 _were analysed using multiplicative analysis of variance (ANOVA), with treatment and country as fixed factors and the pre-administration value (t = -5 minutes) as a covariate. The change in respiratory rate was analysed using additive ANOVA. The proportions of patients with treatment success, treatment failure and reports of effective medication were analysed using logistic regression models with treatment and country as factors. Odds ratios were estimated and described with 95% confidence limits. Clinical laboratory data, vital signs and physical measurements relating to safety were compared between treatment groups with an additive ANOVA model. Adverse-event data were evaluated using primarily descriptive statistics.

## Results

### Patients

Patients were enrolled and treated between April 2002 and August 2003. A total of 277 patients were recruited into the study, 115 of whom fulfilled the inclusion criteria and were randomized to receive treatment with either budesonide/formoterol (n = 58) or formoterol (n = 57). One patient, who was randomized into the formoterol group, discontinued the study as a result of an adverse event. The full analysis set comprised all randomized patients.

The two treatment groups were comparable at baseline, in terms of both demographics and other clinical characteristics (Table 1). Patients reported a background of chronic severe asthma of longstanding duration (median 21 years). In the year before entry into the trial, 96% of the patients had experienced an exacerbation, with a mean number of 6 events per patient per year. Despite this, only 29% of the patients reported using preventative ICS. At study entry, the mean FEV_1 _was 1.13 L (40% of the predicted normal value). After treatment with salbutamol 400 μg, the mean reversibility in FEV_1 _was 5.6% of the baseline value (2.2% of predicted normal).

**Table 1:** Baseline demographics and clinical characteristics

**Characteristic**	**Formoterol (n = 57)**	**Budesonide/formoterol (n = 58)**
Male/female, n	20/37	22/36
Age, years (range)	43.9 (12–72)	45.9 (13–78)
Race, n (%)		
Caucasian	39 (68)	37 (64)
Black	0	1 (2)
Other	18 (32)	20 (34)
Asthma duration, years (range)	20 (1–65)	22.5 (0–54)
Acute severe asthma exacerbations in the 12 months before study entry		
Number (%) of patients with event	56 (98)	54 (93)
Mean number of events (range)	6.3 (1–30)	6.4 (1–40)
Duration of current asthma exacerbation, n		
<6 hours	2	2
6–12 hours	4	1
12–24 hours	4	11
>24 hours	47	43
FEV_1_, L (range)	1.15 (0.7–2.0)	1.12 (0.6–1.9)
FEV_1_, % of predicted normal (range)	41 (30–55)	40 (26–55)
Reversibility, % of predicted normal (range)	2.4 (-8 to +8)	2.1 (-7 to +8)
Reversibility, % of baseline (range)	5.8 (-18 to +23)	5.5 (-16 to +20)
Number (%) of patients prescribed long-acting β_2_-agonist at entry	4 (7)	6 (10)
Number (%) of patients prescribed ICS at entry^a^	16 (28)	17 (29)
ICS at entry, μg (range)	768 (160–2560)	624 (100–2400)

All values are presented as absolute numbers or as means, except asthma duration, for which the median is given. ^a^Dose of inhaled corticosteroid (budesonide equivalents) in patients receiving inhaled corticosteroid. FEV_1 _= forced expiratory volume in 1 second; ICS = inhaled corticosteroids.

### Efficacy

#### Lung function

**Figure 2:**
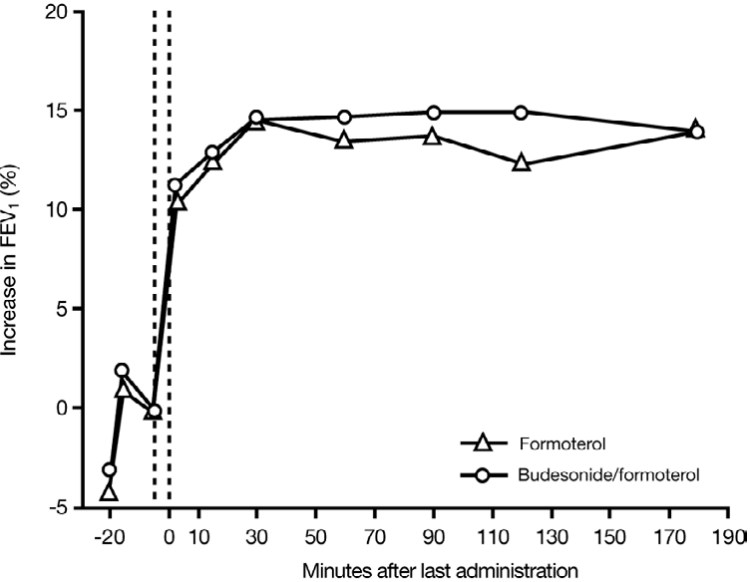
Mean increase in FEV_1 _from baseline in patients treated with either formoterol or budesonide/formoterol. At screening (t = -20 minutes), salbutamol 400 μg was administered to all patients to establish their relative refractoriness to β_2_-agonist therapy. Patients in the formoterol group received formoterol 9 μg, 2 inhalations at t = -5 minutes and 2 inhalations at 0 minutes (total dose 36 μg). Patients treated with budesonide/formoterol received budesonide/formoterol 320/9 μg, 2 inhalations at t = -5 minutes and 2 inhalations at 0 minutes (total dose 1280/36 μg). The timing of study drug administration is shown by vertical dotted lines on the figure. Patients received oral prednisolone 60 mg 90 minutes after the last administration of study drug.

FEV_1 _increased after study drug administration in both treatment groups (Figure 2). The average increase from baseline in FEV_1 _(area under the curve) from the first intake of study drug to the 90-minute measurement was 17.4% in patients receiving budesonide/formoterol and 16.5% in formoterol-treated patients. There was no significant difference between the groups for the primary outcome variable.

There were no statistically significant differences between the budesonide/formoterol and formoterol treatment groups with regard to either the change in FEV_1 _at any of the timepoints analysed over the full 180-minute assessment period, or for the maximal FEV_1 _value achieved (Table 2).

**Table 2:** Treatment comparisons (budesonide/formoterol vs formoterol) for all FEV_1 _outcome variables

	**Mean % change from baseline**^*a*^			
			
**Variable**	**Formoterol (n = 57)**	**Budesonide/formoterol (n = 58)**	**Budesonide/formoterol vs formoterol: mean ratio, % (95% confidence limits)**	**p value**
E_av_	16.5	17.4	100.8 (96.1, 105.7)	0.74
E_max_	26.3	27.2	100.7 (95.1, 106.7)	0.80
E_3_	13.3	14.1	100.7 (96.5, 105.1)	0.75
E_15_	15.8	16.4	100.5 (95.9, 105.3)	0.82
E_60_	17.2	18.6	101.2 (95.8, 106.9)	0.67
E_90_	18.2	19.7	101.3 (95.5, 107.3)	0.67
E_180_	16.5	16.5	100.0 (93.7, 106.7)	1.0

^a^Based on adjusted ratios from an analysis of variance model.

E_av _is the average FEV_1 _(area under the curve) from the first intake of study drug (-5 minutes) to the +90-minute measurement (primary outcome variable). E_max _is the change in FEV_1 _from pre-administration to the maximal value after administration. E_3_, E_15_, E_60_, E_90 _and E_180 _are the changes in FEV_1 _from pre-administration to 3, 15, 60, 90 and 180 minutes after the last administration of the study drug, respectively.

FEV_1 _= forced expiratory volume in 1 second.

#### Respiratory rate

**Figure 3:**
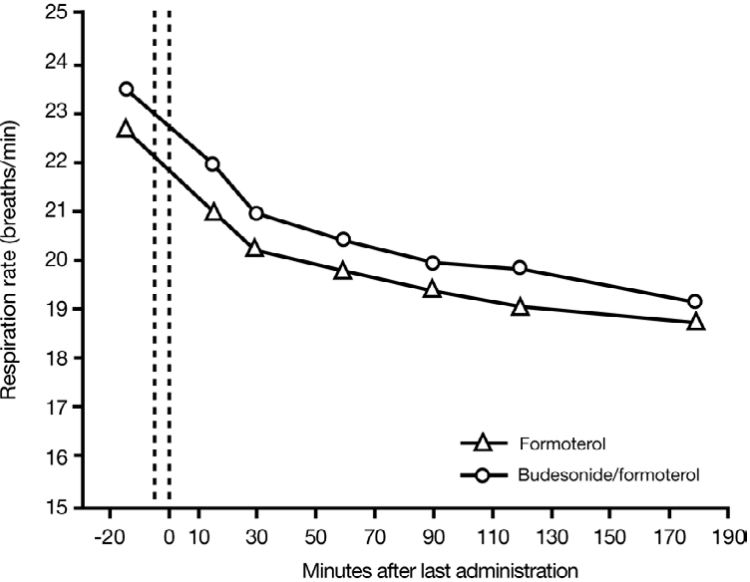
Change in respiratory rate in patients treated with formoterol or budesonide/formoterol. At screening (t = -20 minutes), salbutamol 400 μg was administered to all patients to establish their relative refractoriness to β_2_-agonist therapy. Patients in the formoterol group received formoterol 9 μg, 2 inhalations at t = -5 minutes and 2 inhalations at 0 minutes (total dose 36 μg). Patients treated with budesonide/formoterol received budesonide/formoterol 320/9 μg, 2 inhalations at t = -5 minutes and 2 inhalations at 0 minutes (total dose 1280/36 μg). The timing of study drug administration is shown by vertical dotted lines on the figure. Patients received oral prednisolone 60 mg 90 minutes after the last administration of study drug.

The respiratory rate decreased over time in both treatment groups (Figure 3), with no statistically significant between-group difference being apparent. In patients receiving budesonide/formoterol, the mean pre-dose value (breaths per minute) was 23.5, decreasing to 19.2 at t = 180 minutes. The corresponding values for the formoterol group were 22.6 and 18.4, respectively.

#### Treatment success, treatment failure and effectiveness of medication

There was a slight numerical advantage in the percentage of patients reporting treatment success in the budesonide/formoterol group (79% and 90% at 90 and 180 minutes, respectively) compared with those being treated with formoterol (74% and 84% at 90 and 180 minutes, respectively). Despite the initially poor response to salbutamol in all patients, only a small minority of treatment failures were seen in both groups (10% and 16% for patients treated with budesonide/formoterol and formoterol, respectively). The majority of patients described their medication as effective. After 3 minutes, 97% of patients receiving budesonide/formoterol considered their medication to be effective; this figure remained at 97% at 15 minutes. The corresponding values for the formoterol treatment group were 93% and 96%, respectively.

There were no statistically significant between-group differences for any of these variables.

### Safety

#### Adverse events

Nine patients in each treatment group (16%) reported a total of 24 adverse events. There were 13 adverse events in patients receiving budesonide/formoterol and 11 in the formoterol treatment group. These events were mostly mild or moderate in intensity. Tremor and headache were the most frequently reported adverse events and there were no differences between the treatment groups in the incidence of patients reporting these symptoms (Table 3).

**Table 3:** Most commonly reported adverse events in patients treated with formoterol or budesonide/formoterol

**Preferred term**	**Formoterol (n = 57)**	**Budesonide/formoterol (n = 58)**
Tremor	4 (7)	4 (7)
Headache	3 (5)	3 (5)
Asthma aggravated	1 (2)	1 (2)
Tachycardia	2 (4)	0
AE associated with test procedure	0	1 (2)
T-wave changes	0	1 (2)
Chest pain	0	1 (2)
Hypokalaemia	1 (2)	0
Dizziness	0	1 (2)
Pruritus	0	1 (2)

The number (%) of patients with each adverse event is given.

AE = adverse event.

One patient in the formoterol group discontinued the study after 2 hours because of worsening asthma. A patient in the budesonide/formoterol group suffered a serious deterioration in asthma symptoms but managed to complete the study. These two episodes were reported as serious adverse events (asthma aggravated). Neither was considered to be causally related to the study drug. There were no deaths during the study.

#### Clinical laboratory data and other safety evaluations

Treatment with both budesonide/formoterol and formoterol was well tolerated and systemic effects were similar for both treatments. The majority of patients in both treatment groups had normal serum potassium before treatment and at the end of the study period. The mean values for the average serum potassium during the 180 minutes after study drug administration were 3.82 and 3.88 mmol/L for the budesonide/formoterol and formoterol groups, respectively. The corresponding mean pre-dose values were 3.83 and 4.01 mmol/L, respectively. Abnormally low values (<3.0 mmol/L) were reported for two patients 180 minutes after the last intake of study medication; both of these patients were in the formoterol group. The lowest treatment value recorded for a patient in the budesonide/formoterol group was a transient decrease to 2.7 mmol/L after 90 minutes; the lowest value for formoterol-treated patients was 2.6 mmol/L, which occurred in a patient with a pre-dose value of 2.4 mmol/L. In the formoterol group, one case of hypokalaemia was reported as an adverse event. Overall, the effects of budesonide/formoterol and formoterol on serum potassium were small and were not considered to be clinically important; there were no statistically significant differences between the treatment groups with regard to this parameter.

Vital signs, ECG parameters and oxygen saturation also showed only small changes over time during the course of the study. There were no statistically significant differences between the treatments for the average or maximum/minimum values for any of these variables, with the exception of heart rate, for which budesonide/formoterol had a lower maximum value (mean of 91.6 beats per minute) than formoterol (mean of 94.3 beats per minute; p = 0.026).

## Discussion

Patients with acute asthma require efficacious medication to reduce bronchoconstriction. In this study, budesonide/formoterol and formoterol proved similarly safe and effective for the treatment of patients with acute asthma who had not initially responded adequately to the short-acting β_2_-agonist, salbutamol. There was no difference between the two treatments with regard to their efficacy and tolerability over the 180-minute assessment period. At the end of the study, a high proportion of patients in both groups had been successfully treated.

Previous studies have demonstrated that formoterol is at least as effective as salbutamol for the treatment of acute exacerbations [7,6] and its role in providing as-needed relief from symptoms has been well demonstrated [14,15]. The similar efficacy of budesonide/formoterol and formoterol seen in our patient population suggests that budesonide/formoterol can also be used for as-needed symptom relief in patients with acute severe bronchospasm. Further evidence to support this has been obtained by Balanag and colleagues [16] who demonstrated that budesonide/formoterol and salbutamol had similar short-term efficacy in relieving acute asthma exacerbations. However, unlike the Balanag study, we enrolled patients with acute asthma who demonstrated initial refractoriness to salbutamol. We were, however, unable to demonstrate evidence of an enhanced β_2_-receptor response in patients receiving therapy with ICS, as has been described previously [10,11,17]. A possible reason for our results is that, despite our patients' poor response to salbutamol 400 μg (mean improvement in FEV_1 _of only 5.6% from baseline), they may not have been sufficiently refractory to demonstrate the potential benefit provided by ICS. This is suggested by the rather good initial responses (additional improvements in FEV_1 _of 13.3% and 18.2% at 3 minutes and 90 minutes after dosing, respectively) in the patients who received formoterol alone.

The identification of patients with refractoriness to β_2_-agonists in acute asthma is complicated by both conceptual and methodological problems. Among the former are the difficulty in distinguishing between β_2_-agonist refractoriness caused by downregulation – a feature that has been confirmed *in vitro *[8], but not *in vivo *– and a lack of clinical response caused by pathological changes in asthmatic airways (e.g. mucus plugging, oedema of the bronchial wall) and chronic structural changes (such as airway remodelling) [18,19]. Assays of β_2_-agonist receptor density on circulating monocytes, together with other *in vitro *tests, may have been useful, but the results of such testing would not have been available in time to assist with patient selection for this acute study. A minority of patients were being treated with ICS at the time of their exacerbation, but most had a history of previous exacerbations, suggesting that many patients' asthma had been chronically undertreated. In addition, for 78% of the patients the acute symptoms had lasted for >24 h before study entry and so it is possible that, in many patients, airway pathology may have been well established. Consequently, airway pathology, rather than downregulation of β_2_-agonist receptors, may have accounted for β_2_-agonist refractoriness.

Another challenge when selecting patients for a study such as the one we have reported here, is to establish accurately patients' prior dosing with β_2_-agonists. Patient recall of β_2_-agonist use is often poor. Moreover, we waited only 15 minutes after administering salbutamol 400 μg before patients were treated with the study drug. This was necessary because patients were distressed and it was considered neither safe nor compassionate to await the peak effect of the salbutamol (between 15 and 30 minutes after administration) to exclude patients who responded more slowly. In contrast, patients were followed for longer periods after receiving the study drugs. There was, however, some evidence that the initial response to salbutamol was already at a plateau or in decline when the study drugs were administered, although there was no evidence of a loss of effect with either of the study drugs during the 3-hour assessment (Figure 2).

Differences in methods of patient selection may have accounted for the differences between our results and those reported with early ICS dosing in previous trials [10,11,17]. However, it should be noted that Pansegrouw [11] provided little detail from which to gauge his patients' prior treatment or degree of refractoriness to β_2_-agonists, although the patients in that study had asthma of a similar severity to the patients enrolled into our study.

There are other possible reasons for a lack of additional benefit with ICS. For instance, if patients had received previous treatment with corticosteroids, there might have been a carry-over of the effect of this medication. Although patients were not permitted to receive ICS within the 8 hours before the baseline measurements, or oral/systemic steroids in the 48 hours before the baseline measurements, a protective effect of prior dosing may still have been apparent. However, as only 29% of the patients were using ICS on study entry, this may not account for the lack of additional benefit seen with budesonide/formoterol compared with formoterol in this study. The early response to ICS may depend on the repeated administration of high doses of the drug, as postulated by Rodrigo and Rodrigo [17]. In their study, patients with acute asthma of more than 24 hours' duration had a significantly better response when treated with high cumulative doses of flunisolide in addition to salbutamol than those treated with salbutamol alone. Patients in our study received only two doses of budesonide/formoterol, which may not have been sufficient for a meaningful topical response. Finally, there are limitations to using spirometry alone in determining nonresponsiveness to β-agonists.

Pansegrouw [11] is not alone in demonstrating the effect of ICS in refractory asthma. Aziz and Lipworth [10] reported that a bolus of inhaled budesonide (1600 μg) rapidly reversed formoterol subsensitivity to adenosine monophosphate bronchoprotection in patients with asthma who were receiving regular formoterol. Additionally, other mechanisms of action of ICS in acute asthma have been proposed. Engel and colleagues [20] demonstrated improvements in lung function over 3 hours with budesonide administered alone (i.e. in the absence of β_2_-agonists) versus placebo. While an improvement in lung function was evident 1 hour after drug administration, the effects increased over time to become statistically significantly different compared with placebo at 3–4 hours [20]. In the present study, efficacy data were collected over a 180-minute time period. Measurements extending beyond this timeframe may have proved valuable in assessing the contribution of budesonide to improving lung function. However, for ethical reasons all patients were treated with oral prednisolone 90 minutes after receiving the last dose of study medication, and this may have limited the utility of observations continuing for longer than 3 hours.

It is also possible that the dose of budesonide used in this study was too low. In patients who deteriorate whilst on regular doses of ICS, doubling the maintenance dose when symptoms of an exacerbation become apparent has not been shown to be effective in preventing the exacerbation [21,22], but a fourfold increase in dose at the onset of an exacerbation may be beneficial [23]. These studies [21-23] examined the effects of treatment on patients over weeks and months; the study that we describe here assessed the efficacy of treating exacerbations over a 180-minute time period. Studies involving budesonide/formoterol for maintenance and reliever therapy have shown that timely increases in the budesonide/formoterol dose as the symptoms of loss of control of asthma become apparent can result in a marked reduction in exacerbations [24,25]. These results suggest that treatment with an ICS and long-acting β_2_-agonist combination (at the dose administered in the present study) is effective in preventing severe exacerbations if given very early, when the symptoms of loss of asthma control first become apparent, but may be less beneficial for patients in whom an exacerbation has become established. In our study investigating symptom relief in patients with acute asthma, the long-acting β_2_-agonist alone was as effective as the combination therapy – even in patients presenting for emergency treatment who had used their short-acting β_2_-agonist liberally and appeared to be refractory to β_2_-agonist therapy, as judged by poor bronchodilatory response to short-acting β_2_-agonists.

In addition to being effective, both budesonide/formoterol and formoterol were safe and well tolerated in the present study. The patterns of adverse events raised no safety concerns and, overall, the changes over time in systemic effects were very minor. These results are in line with those from earlier studies. Ankerst and colleagues [26] have reported systemic effects associated with the administration of budesonide/formoterol to be small, and the systemic effects of formoterol have generally been reported as minor and similar to salbutamol [6,7,16,27].

In conclusion, budesonide/formoterol and formoterol provide rapid relief of acute bronchoconstriction in patients with asthma, producing effects of a similar magnitude. Both preparations are well tolerated.
